# Au^III^ Acyclic (Amino)(N-Pyridinium)carbenoids: Synthesis via Addition of 2-PySeCl to Au^I^-Bound Isonitriles, Structures, and Cytotoxicity

**DOI:** 10.3390/ijms26020483

**Published:** 2025-01-08

**Authors:** Olga V. Repina, Alexey S. Kubasov, Anna V. Vologzhanina, Alexander V. Borisov, Ilya S. Kritchenkov, Ksenia M. Voroshilkina, Alexey A. Nazarov, Dmitriy M. Shchevnikov, Mariya V. Grudova, Rosa M. Gomila, Antonio Frontera, Valentine G. Nenajdenko, Andreii S. Kritchenkov, Alexander G. Tskhovrebov

**Affiliations:** 1 Research Institute of Chemistry, Peoples’ Friendship University of Russia, 6 Miklukho-Maklaya Street, 117198 Moscow, Russiailya.kritchenkov@gmail.com (I.S.K.); shchevnikov.dm@gmail.com (D.M.S.); platinist@mail.ru (A.S.K.); 2 Kurnakov Institute of General and Inorganic Chemistry, 31 Leninsky Prosp., 119991 Moscow, Russia; 3 Nesmeyanov Institute of Organoelement Compounds, Russian Academy of Sciences, Vavilova St. 28, 119334 Moscow, Russia; vologzhanina@mail.ru; 4 Institute of Chemistry, R.E. Alekseev Nizhny Novgorod State Technical University, Minin St. 24, 603155 Nizhny Novgorod, Russia; avb1955@rambler.ru; 5 Department of Chemistry, M.V. Lomonosov Moscow State University, 1 Leninskie Gory, 119991 Moscow, Russia; 6 Departament de Química, Universitat de les Illes Balears, 07122 Palma de Mallorca, Spain; rosa.gomila@uib.es (R.M.G.); toni.frontera@uib.es (A.F.); 7 Branch of Petersburg Nuclear Physics Institute Named by B.P. Konstantinov of National Research Centre «Kurchatov Institute»—Institute of Macromolecular Compounds, Bolshoi pr. VO 31, 199004 St. Petersburg, Russia

**Keywords:** selenazoles, carbenes, isonitriles, gold, non-covalent interactions, halogen bonding, chalcogen bonding

## Abstract

In this study, we report the first example of acyclic (amino)(N-pyridinium)carbenoid gold(III) complexes synthesized via a coupling reaction between 2-pyridylselenyl chloride and Au(I)-bound isonitriles. The reaction involves an initial oxidative addition of the Se–Cl moiety to Au(I), followed by the nucleophilic addition of the pyridine fragment to the isonitrile’s C≡N bond, furnishing a metallacycle. Importantly, this is the first example of the pyridine acting as a nucleophile towards metal-bound isonitriles. Arguably, such an addition is due to the chelate effect. The structures of the gold(III) carbenoid complexes were unambiguously established using X-ray diffraction and NMR spectroscopy. Theoretical calculations, including DFT, Natural Resonance Theory (NRT), and Meyer bond order (MBO) analyses, were used to analyze the different resonance forms. The reaction mechanism was further elucidated using DFT calculations, which identified the oxidative addition as the rate-determining step with a barrier of 29.7 kcal/mol. The nucleophilic addition proceeds with a minimal barrier, making the reaction highly favorable. The antiproliferative activity of new compounds **2a**–**2e** was tested against two human cancer cell lines: A2780 ovarian adenocarcinoma and the A278Cis cisplatin-resistant variant.

## 1. Introduction

**Scheme 1:**
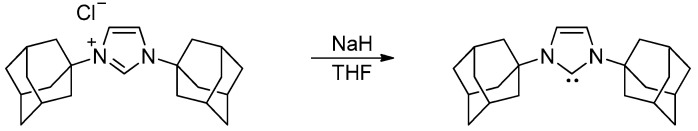
Synthesis of isolable NHC by Arduengo.

Nitrogen-stabilized carbene complexes are of paramount importance in organometallic chemistry, with applications in catalysis, the development of novel antitumor agents, etc. [1,2,3]. N-heterocyclic carbenes (NHCs) currently represent one of the most applicable classes of ancillary ligands in catalysis [4,5]. These carbenes are stable in the free state and were first isolated by Arduengo in 1991 (Scheme 1) [6].

These ligands are particularly significant due to their prominent role in organometallic chemistry and catalysis, where they have emerged as strong competitors to the widely employed phosphine ligands. Many of their complexes serve as catalysts of choice in various organic transformations. Additionally, their electronic and geometric properties can be modified more easily than those of phosphines, offering greater versatility and tunability in catalytic applications.

The most popular NHCs are typically derived from imidazolium salts and, to a lesser extent, imidazolidinium salts. These carbenes are generated through the action of strong bases on the respective salts. Numerous methods have been developed for synthesizing various precursors of NHCs [7]. The significance of this class of ligands has driven extensive research on diverse approaches to the synthesis of their precursors. While these methods are not detailed in this work, they are comprehensively summarized in several review papers [7,8,9,10,11,12,13].

In contrast, acyclic diaminocarbenes (ADCs) have received less attention. Importantly, ADCs have demonstrated their ability to act as strong σ-donor ligands. In 2002, Herrmann showed that ADC ligands induce greater electron density at Rh(I) compared to both saturated and unsaturated five-membered NHCs, as evidenced by the *ν*(CO) values of the square planar Rh(I) carbonyl complexes [14].

However, ADC complexes have one significant advantage over NHC complexes, which is their synthesis via nucleophilic addition to metal-activated isonitriles [15,16,17,18,19,20,21,22,23,24,25]. This route unlocks structures with easily variable steric and electronic properties.

Gold(I) and (III) have shown remarkable efficacy in promoting nucleophilic addition to isonitriles producing ADC complexes [26,27,28,29,30]. The latter has shown promise in catalysis and antiproliferative activity, which is widely being explored currently [31,32,33,34,35,36,37,38,39,40,41,42,43,44,45,46].

Here, we describe the addition of 2-pyridylselenyl chloride to gold(I)-bound isonitriles producing acyclic (amino)(N-pyridinium)carbenoid gold(III) complexes. This is the first example of pyridine acting as a nucleophile in metal-mediated addition to isonitriles. Moreover, novel structures were tested against two human cancer cell lines.

## 2. Results and Discussion

**Scheme 2:**
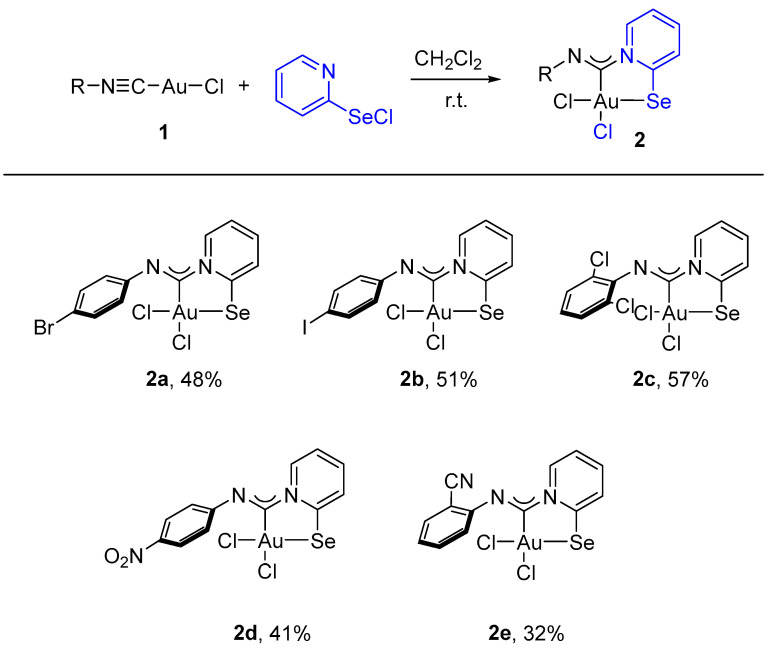
Synthesis of **2a**–**2e**.

For this study, we used Au(I) isocyanide complexes, which were obtained in situ by reacting the corresponding isocyanides with [(Me_2_S)AuCl] in dichloromethane. The addition of 2-pyridylselenyl chloride to solutions of Au(I) isocyanide complexes in CH_2_Cl_2_ resulted in a gradual formation of orange precipitates (Scheme 2). The isolation and analysis of the solids suggested the formation of the adducts of 2-pyridylselenyl chloride with isocyanide-gold(I) chloride in 32–57% yields (Scheme 2).

Complexes **2a**–**2e** are stable at room temperature and exhibit very poor solubility in most organic solvents. However, they are moderately soluble in highly polar solvents such as DMF or DMSO, which allows their characterization by the NMR technique. The ^13^C NMR spectra of **2a**–**2e** (see Section 3) exhibited new characteristic peaks in the range of 163–153 ppm, indicating carbenoid species formation.

**Scheme 3:**
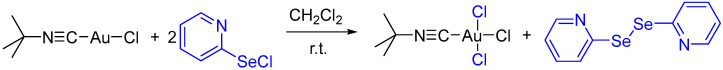
Oxidation of *tert*-butyl isocyanide gold(I) chloride to gold(III) trichloride by 2-PySeCl.

Interestingly, aliphatic isocyanide gold(I) complexes did not produce carbenoid species like **2a**–**2e** upon reaction with 2-PySeCl. The addition of 2-PySeCl to *tert*-butyl isocyanide gold(I) chloride resulted in the formation of isocyanide Au(III) trichloride and 2,2′-dipyridyl diselenide as products (Scheme 3). In this case, 2-PySeCl acted as a chlorinating agent.

**Figure 1:**
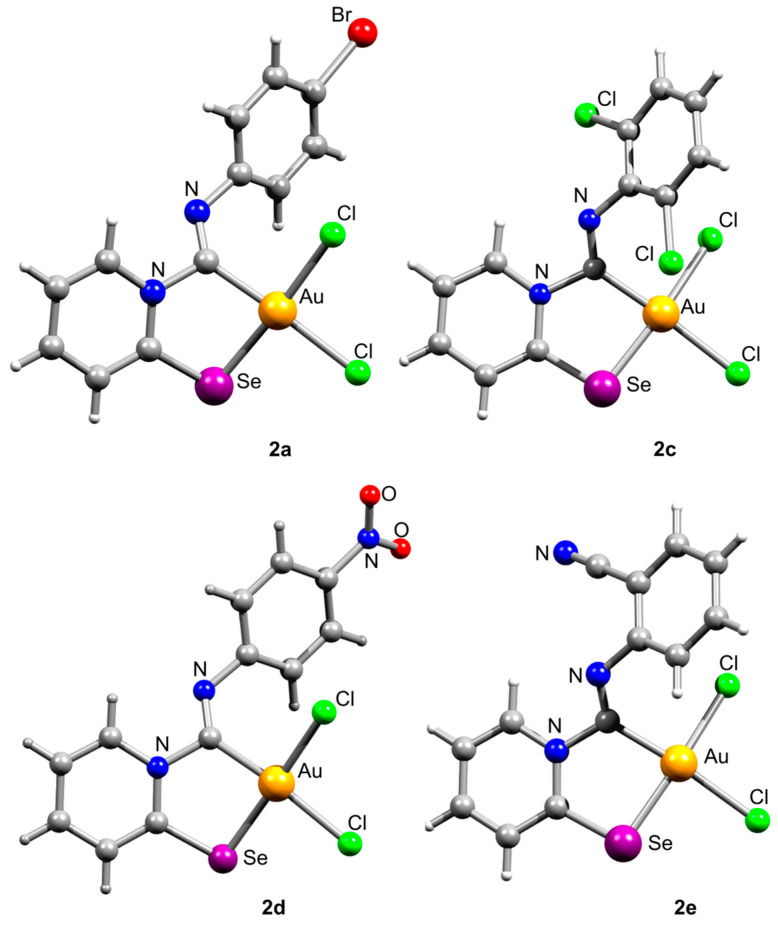
Ball-and-stick representations of crystal structures of **2a** and **2c**–**2e**. Gray and light-gray spheres represent carbon and hydrogen, respectively.

The carbenoid complexes **2a** and **2c**–**2e** could be recrystallized from dichloromethane to produce crystals suitable for X-ray single-crystal analysis, which confirmed the formation of Au(III) carbenoid complexes (Figure 1, Tables S1–S13 and Figures S1–S4).

Single crystals of **2e** contain dichloromethane molecules, while the other complexes crystallize without a solvent. The asymmetric unit of **2c** contains two independent molecules. For all complexes, Au(III) achieves a distorted square geometry with a twisted disposition of two chlorine and C, Se atoms (Figure 1, Table 1). The Au–C distance is the shortest, and the Au–Se distance is the longest.

**Scheme 4:**
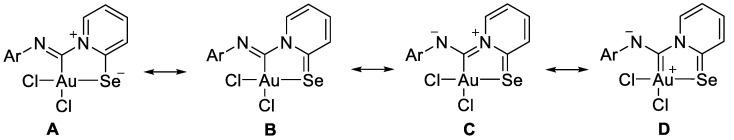
Mesomeric forms for Au^III^ carbenoid complexes **2a**–**2e**.

The structures of **2a**–**2e** could be described by the mesomeric forms **A**–**D** (Scheme 4).

The bond distances in the ligand demonstrate that the main mesomeric form for 2-pyridine-selenyl carbene complexes is mesomeric form **A** with single C–Se and double N=C(carbenoid) bonds. The length of the C–N_Ph_ bond is affected by electronic effects from substituents at different positions in the phenyl ring. The ligand chelates the gold(III) atom with the formation of a rigid five-membered cycle; however, the angle between the pyridine ring and the mean plane formed by the AuCl_2_ fragment varies from 20.1(2) to 36.50(13). The angle between an aryl ring and the pyridine cycle is equal to 15.3(3)–60.4(10) due to rotation around a single N–C bond.

**Figure 2:**
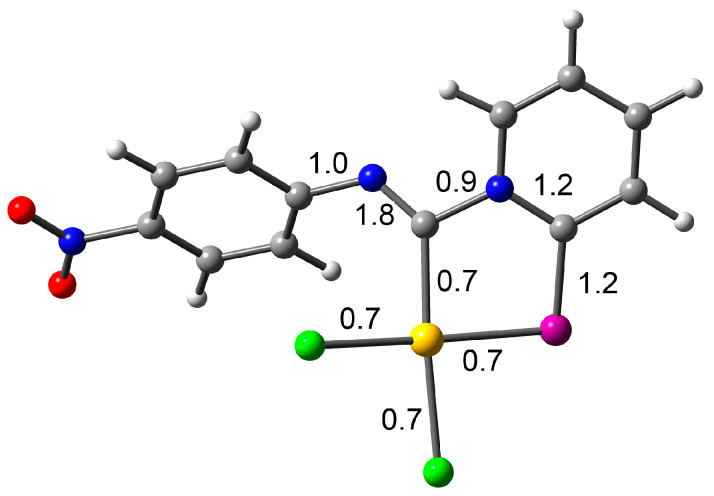
RI-BP86-D4/def2-TZVP optimized geometry of compound **2d**, indicating the Meyer bond order.

To gain a deeper insight into which mesomeric forms (Scheme 4) contribute most to the final structure, we performed additional theoretical calculations. Specifically, the percentage of each form was computed using Natural Resonance Theory (NRT) within the Natural Bond Orbital (NBO) framework, along with Meyer bond order (MBO) values. The NRT results indicate negligible contributions from forms **C** and **D**, while form **A** dominates, with a 70% contribution. This aligns well with the MBO analysis (see Figure 2), which shows that the Npy–C(Au) bond has a bond index below 1, effectively ruling out any significant contribution from resonance form **C**, which involves a Npy=C(Au) double bond. The MBO values for all coordination bonds are approximately 0.7, typical of coordination bonds with bond orders less than 1, further excluding any double bond character between the gold and carbon atoms, thereby dismissing resonance form **D**. Additionally, the MBO value of nearly 2 for the Nph–C(Au) bond is consistent with forms **A** and **B**, confirming no contribution from forms **C** or **D**. Finally, the MBO value of 1.2 for both the C–Se and pyridine C–N bonds suggests some contribution from resonance form **B**, corroborated by NRT calculations.

Although the nucleophilic addition to the C≡N triple bond of metal-bound isonitriles is well documented and widely studied in the context of the synthesis of metal carbene complexes and their applications, this is the first example of pyridine functioning as a nucleophile in a metal-mediated coupling with isonitriles. As a result, this represents the first example of acyclic (amino)(N-pyridinium)carbenoid metal complexes.

**Figure 3:**
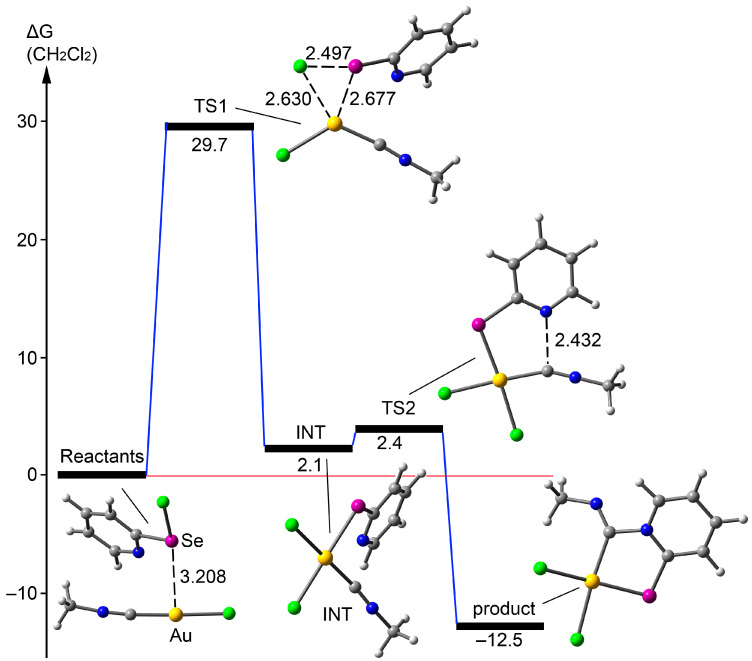
Energetic profile for the reaction of [(Me_2_S)AuCl] with CH_3_-N≡C–Au–Cl at the RI-BP86-D4(CH_2_Cl_2_)/def2-TZVP level of theory. Distances are shown in Å.

The mechanism of the new coupling reaction between 2-pyridylselenyl chloride and Au(I) isocyanide complexes formally involves two key steps: the oxidative addition of the Se–Cl moiety to Au(I) and the nucleophilic addition of the pyridine fragment to the isonitrile’s CN triple bond. These steps may occur either sequentially or simultaneously. To elucidate the mechanism, we performed additional DFT calculations, which ruled out the simultaneous mechanism. For computational efficiency, we simplified the isocyanide complex to CH_3_-N≡C–Au–Cl. We explored the potential energy surface connecting the [(Me_2_S)AuCl] and CH_3_-N≡C–Au–Cl reactants to the final adduct using the NEB-TS (Nudged Elastic Band with TS optimization) method, which locates the transition state (TS) by using the geometries of the reactants and products. The NEB-TS analysis identified an intermediate corresponding to the oxidative addition of the Se–Cl moiety to Au(I) (Figure 3). Starting from this intermediate, we computed two transition states, linking it to both the reactants and the product. The ΔG values in CH_2_Cl_2_ reveal that the oxidative addition is the rate-determining step, with a barrier of 29.7 kcal/mol. Interestingly, the reactants form a supramolecular complex prior to the oxidative addition, where the Se atom is preorganized 3.028 Å from the Au atom. The second step, the nucleophilic addition of the pyridine fragment to the CN bond, occurs with a minimal barrier of 0.3 kcal/mol, leading to the final product, which is 12.5 kcal/mol more stable than the starting material.

Gold carbene complexes are known for their anticancer activity. The antiproliferative activity of new compounds **2a**–**2e** was tested against two human cancer cell lines, A2780 ovarian adenocarcinoma and the A278Cis cisplatin-resistant variant, using the standard MTT colorimetric assay. Cisplatin was used as a control in the study. The new compounds **2a**–**2e** demonstrated high antiproliferative activity in the low micromolar range. They were about 3–5 times more potent than cisplatin against the cisplatin-sensitive cell line and about 5–6 times more potent against the cisplatin-resistant variant (Table 2).

Among the new compounds, the one with the nitro group **2d** was found to be the most active against both cancer cell lines. When comparing the antiproliferative activity of the new compounds against the A2780 and A2780 cis cell lines, it was found that the complexes were less potent against cisplatin-resistant cells (Table 2, Rf); however, the resistant index was lower compared to the cisplatin. These results suggest that the new compounds may have a different mode of action compared to cisplatin, which will be studied in more details in the future.

## 3. Methods

### 3.1. Experimental Details

**General remarks**. No uncommon hazards were noted as stemming from the experimental work carried out. All manipulations were carried out in air. All the reagents used in this study were obtained from commercial sources (Aldrich, TCI-Europe, Strem, ABCR). Commercially available solvents were purified by conventional methods and distilled immediately prior to use. Mass spectra were recorded on a Bruker micrOTOF spectrometer equipped with an electrospray ionization (ESI) source; MeOH or MeCN was used as a solvent. NMR spectra were recorded on a Bruker Avance neo 700; chemical shifts (*δ*) are given in ppm, and coupling constants (*J*) are given in Hz. IR spectra were recorded on a Shimadzu Inspirit IR–Fourier spectrometer equipped with the QATAR-Singapore attenuated total reflectance sample holder.

**Synthetic part.**


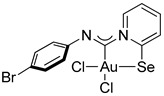


**Compound 2a**. **1** [(Me_2_S)AuCl] (15 mg, 0.05 mmol) was added to a solution of *p*-bromophenyl isocyanide (9 mg, 0.05 mmol) in CH_2_Cl_2_ (2 mL), and the solution was stirred for 15 min. After that, a solution of 2-PySeCl (10 mg, 0.05 mmol) in CH_2_Cl_2_ (3 mL) was added to the mixture, which was stirred for 4 h. An orange precipitate formed, which was filtered off and washed with MeOH (3 mL) and Et_2_O (3 mL) and dried under a vacuum. Yield: 15 mg, 48%. ^1^H NMR (400 MHz, DMSO-*d*_6_) *δ* 9.33 (d, *J* = 6.6 Hz, 1H), 8.34 (d, *J* = 8.2 Hz, 1H), 8.26 (t, *J* = 7.7 Hz, 1H), 7.74 (t, *J* = 6.9 Hz, 1H), 7.62 (d, *J* = 8.5 Hz, 2H), 7.14 (d, *J* = 8.5 Hz, 2H). ^13^C NMR (101 MHz, DMSO) δ 154.4, 146.5, 146.1, 144.4, 142.3, 131.7, 127.6, 123.3, 122.9, 119.0. MS (ESI^+^): found, 606.8143 [M]^+^; calcd for C_12_H_8_AuBrCl_2_N_2_Se, 606.8151. Crystals, suitable for X-ray analysis, were obtained directly from the reaction mixture.


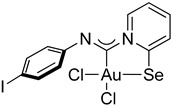


**Compound 2b** [(Me_2_S)AuCl] (15 mg, 0.05 mmol) was added to a solution of *p*-iodophenyl isocyanide (12 mg, 0.05 mmol) in CH_2_Cl_2_ (2 mL), and the solution was stirred for 15 min. A white precipitate formed. After that, a solution of 2-PySeCl (10 mg, 0.05 mmol) in CH_2_Cl_2_ (3 mL) was added to the mixture, which caused the white precipitate to dissolve, and the mixture was stirred for 4 h. An orange precipitate formed, which was filtered off and washed with MeOH (3 mL) and Et_2_O (3 mL) and dried under a vacuum. Yield: 20 mg, 51%. ^1^H NMR (400 MHz, DMSO-*d6*) *δ* 9.32 (d, *J* = 6.3 Hz, 1H), 8.34 (d, *J* = 8.2 Hz, 1H), 8.25 (t, *J* = 7.7 Hz, 1H), 7.81–7.68 (m, 3H), 7.00 (d, *J* = 8.5 Hz, 2H). ^13^C NMR (101 MHz, DMSO-*d6*) *δ* 154.3, 146.5, 145.9, 144.7, 142.3, 138.6, 137.5, 127.6, 123.4, 122.9. MS (ESI^+^): found, 654.8017 [M]^+^; calcd for C_12_H_8_AuICl_2_N_2_Se, 654.8013.


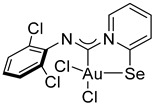


**Compound 2c** [(Me_2_S)AuCl] (15 mg, 0.05 mmol) was added to a solution of 2,6-dichlorophenyl isocyanide (9 mg, 0.05 mmol) in CH_2_Cl_2_ (2 mL), and the solution was stirred for 15 min. After that, a solution of 2-PySeCl (10 mg, 0.05 mmol) in CH_2_Cl_2_ (3 mL) was added to the mixture, which was stirred for 4 h. The reaction mixture was left overnight, and the residue was washed with MeOH (3 mL) and Et_2_O (3 mL) and dried under a vacuum. Yield: 17 mg, 57%. ^1^H NMR (700 MHz, DMSO-*d6*) *δ* 9.10 (d, *J* = 6.7, 1H), 8.41 (d, *J* = 8.0 Hz, 1H), 8.33–8.29 (m, 1H), 7.79 (td, *J* = 7.1, 1.2 Hz, 1H), 7.53 (d, *J* = 8.2 Hz, 2H), 7.25 (t, *J* = 8.1 Hz, 1H). ^13^C NMR (176 MHz, DMSO-*d6*) *δ* 155.79, 153.30, 147.16, 141.32, 140.17, 128.44, 128.01, 126.94, 124.61, 123.40. MS (ESI^+^): found, 560.8614 [M-Cl]^+^; calcd for C_12_H_7_AuCl_3_N_2_Se, 560.8506. Crystals, suitable for X-ray analysis, were obtained from the reaction mixture.


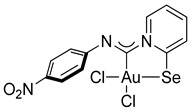


**Compound 2d** [(Me_2_S)AuCl] (15 mg, 0.05 mmol) was added to a solution of *p*-nitrophenyl isocyanide (8 mg, 0.05 mmol) in CH_2_Cl_2_ (2 mL) and then stirred for 15 min. A white precipitate formed. After that, a solution of 2-PySeCl (10 mg, 0.05 mmol) in CH_2_Cl_2_ (3 mL) was added to the mixture, which caused the white precipitate to dissolve, and the mixture was stirred for 4 h. The reaction mixture was left overnight, and the residue was washed with MeOH (3 mL) and Et_2_O (3 mL) and dried under a vacuum. Yield: 12 mg, 41%. ^1^H NMR (400 MHz, DMSO-*d6*) *δ* 9.36 (d, *J* = 6.5 Hz, 1H), 8.38 (d, *J* = 8.2 Hz, 1H), 8.33–8.23 (m, 3H), 7.76 (t, *J* = 6.9 Hz, 1H), 7.39 (d, *J* = 8.9 Hz, 2H). ^13^C NMR (101 MHz, DMSO-*d6*) *δ* 155.0, 150.6, 148.0, 146.8, 144.9, 142.5, 127.60, 124.6, 122.9, 121.8. MS (ESI^+^): found, 573.8892 [M]^+^; calcd for C_12_H_9_AuCl_2_N_3_O_2_Se, 573.8897. Crystals, suitable for X-ray analysis, were obtained from the reaction mixture.


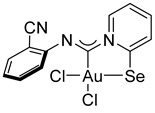


**Compound 2e** [(Me_2_S)AuCl] (15 mg, 0.05 mmol) was added to a solution of 2-cyanophenyl isocyanide (7 mg, 0.05 mmol) in CH_2_Cl_2_ (2 mL) and then stirred for 15 min. After that, a solution of 2-PySeCl (10 mg, 0.05 mmol) in CH_2_Cl_2_ (3 mL) was added to the mixture, which was stirred for 4 h. The reaction mixture was left overnight, and the residue was washed with MeOH (3 mL) and Et_2_O (3 mL) and dried under a vacuum. Yield: 9 mg, 32%. ^1^H NMR (400 MHz, DMSO-*d6*) *δ* 9.11 (d, *J* = 6.4 Hz, 1H), 8.39 (d, *J* = 8.2 Hz, 1H), 8.34–8.24 (m, 1H), 7.87 (d, *J* = 8.8 Hz, 1H), 7.82 (t, *J* = 6.9 Hz, 1H), 7.75 (t, *J* = 8.5 Hz, 1H), 7.43 (t, *J* = 7.7 Hz, 1H), 7.36 (d, *J* = 7.9 Hz, 1H).^13^C NMR (101 MHz, DMSO-*d6*) *δ* 155.6, 150.5, 147.7, 146.9, 141.5, 134.0, 132.7, 127.8, 126.6, 123.3, 121.5, 117.1, 103.9. MS (ESI^+^): found, 553.8992 [M]^+^; calcd for C_13_H_8_AuCl_2_N_3_Se, 553.8999. Crystals, suitable for X-ray analysis, were obtained from the reaction mixture.

#### Reaction of [(tBuNC)AuCl] with 2-PySeCl

[(Me_2_S)AuCl] (15 mg, 0.05 mmol) was added to a solution of *tert*-butyl isocyanide (4 mg, 0.05 mmol) in CH_2_Cl_2_ (2 mL) and the solution was stirred for 15 min. After that, a solution of 2-PySeCl (20 mg, 0.1 mmol) in CH_2_Cl_2_ (3 mL) was added to the mixture and stirred overnight. Then, the reaction mixture was concentrated under a vacuum and separated by column chromatography (eluent: DCM) to give white crystals of [(*t*BuNC)AuCl_3_] and yellow crystals of Py_2_Se_2_. Yield: 15 mg (74%) and 15 mg (94%), respectively.

### 3.2. Theoretical Methods

The structures and relative energies of all systems considered in the mechanistic analysis were optimized at the PBE0-D4/def2-TZVP level of theory using ORCA 5.1 [47]. The hybrid functional PBE0 [48], corrected for dispersion effects with the D4 method [49], was employed alongside a triple-ζ quality basis set [50]. Frequency calculations confirmed the stationary points, ensuring they correspond to minima or transition states. To locate the transition states, the Nudged Elastic Band (NEB) method available in ORCA was applied. This approach identifies a minimum energy pathway by constructing a series of atomic configurations, known as “images” that link the reactant and product states. A detailed explanation of the NEB implementation can be found in the study by Ásgeirsson et al. [51].

## 4. Conclusions

The reaction between the isonitrile gold(I) complex and 2-pyridylselenyl chloride resulted in the formation of unprecedented (amino)(N-pyridinium)carbenoid gold(III) derivatives. The addition proceeds via two key steps: the oxidative addition of Se–Cl moiety to the Au(I) and the nucleophilic addition of the pyridine group to the CN triple bond of metal-bound isonitrile. Importantly, this is a first example of pyridine acting as a nucleophile in the reaction with the CN bond of isonitrile. Arguably, such an addition is due to the chelate effect. The reaction mechanism was elucidated using DFT calculations, which identified the oxidative addition as the rate-determining step with a barrier of 29.7 kcal/mol.

Moreover, we tested the new compounds **2a**–**2e** against two human cancer cell lines, A2780 ovarian adenocarcinoma and the A278Cis cisplatin-resistant variant, and the new compounds were about 3–5 times more potent than cisplatin against the cisplatin-sensitive cell line and about 5–6 times more potent against the cisplatin-resistant variant.

## Figures and Tables

**Table 1:** Selected interatomic distances and angles (Å, °) in **2a** and **2c**–**2e**.

Parameter	2a	2c	2d	2e
Au−Cl	2.244(2)–2.357(2)	2.330(3)–2.354(3)	2.3371(14)–2.3427(13)	2.333(2)–2.349(2)
Au−Se	2.4060(9)	2.3699(15)–2.3866(15)	2.3910(6)	2.3878(16)
Au−C	2.021(9)	1.983(13)–2.024(13)	2.020(5)	2.022(6)
C–Se	1.884(9)	1.869(13)–1.873(14)	1.881(5)	1.877(6)
N=C	1.239(12)	1.267(18)–1.285(18)	1.247(7)	1.257(7)
C–N_Py_	1.481(10)	1.473(14)–1.493(15)	1.472(7)	1.473(7)
C–N_Ph_	1.427(11)	1.368(16)–1.372(16)	1.421(7)	1.403(7)
Cl–Au–Cl	89.93(7)	91.5(1)–93.6(1)	91.84(5)	91.78(9)
C–Au–Se	83.2(2)	83.6(4)–86.1(4)	84.47(15)	84.31(17)
α1 ^a^	15.3(3)	24.3(5)–26.3(5)	52.3(8)	60.4(10)
α2 ^b^	36.50(13)	20.1(2)–27.1(2)	30.78(9)	35.42(11)

^a^ The fold angle between the pyridine and phenyl cycles. ^b^ The fold angle between the 2-pyridylselenyl and the AuCl_2_ fragments.

**Table 2:** Antiproliferative activity of 2-pyridine-selenyl gold (III) carbene complexes **2a**–**e** and cisplatin against human cancer cells. R_f_ (resistance factors) showed the resistance coefficient. The results are the mean values ± SD of three independent experiments, each of which was performed in triplicate.

Compounds	IC_50_ (72 h)/μM		
	A2780	A2780Cis	R_f_
**2a**	0.70 ± 0.07	2.87 ± 0.08	4.1
**2b**	0.97 ± 0.13	2.59 ± 0.03	2.7
**2c**	1.09 ± 0.04	2.91 ± 0.19	2.7
**2d**	0.52 ± 0.07	2.47 ± 0.11	4.7
**2e**	0.71 ± 0.06	2.65 ± 0.17	3.7
**Cisplatin**	2.64 ± 0.35	15.6 ± 2.1	5.9

## Data Availability

Data are contained within the article and Supplementary Materials.

## Supplementary Materials

The following supporting information can be downloaded at: www.mdpi.com/article/10.3390/ijms26020483/s1.
