# Supplementary material for: AuIII Acyclic (Amino)(N-Pyridinium)carbenoids: Synthesis via Addition of 2-PySeCl to AuI-Bound Isonitriles, Structures, and Cytotoxicity
Source: Int J Mol Sci. 2025 Jan 8;26(2):483. doi: 10.3390/ijms26020483 (PMC11765275; doi:10.3390/ijms26020483)
Supplement: Supplementary file 1 [file ijms-26-00483-s001.zip › ijms-3383716-supplementary.pdf]

Supplementary Materials

Au^{III} Acyclic (Amino)(N-Pyridinium)carbenoids: Synthesis via Addition of 2-PySeCl to Au^{I}-Bound Isonitriles, Structures, and Cytotoxicity

Olga V. Repina [1], Alexey S. Kubasov [2], Anna V. Vologzhanina [3], Alexander V. Borisov [4], Ilya S. Kritchenkov [1], Ksenia M. Voroshilkina [5], Alexey A. Nazarov [5], Dmitriy M. Shchevnikov [1], Mariya V. Grudova [1], Rosa M. Gomila [6], Antonio Frontera [6], Valentine G. Nenajdenko [5,*], Andreii S. Kritchenkov [1,7] and Alexander G. Tskhovrebov [1,*]

[1] Peoples' Friendship University of Russia, 6 Miklukho-Maklaya Street, 117198 Moscow, Russia
[2] Kurnakov Institute of General and Inorganic Chemistry, 31 Leninsky Prosp., 119991 Moscow, Russia
[3] Nesmeyanov Institute of Organoelement Compounds, Russian Academy of Sciences, Vavilova St. 28, 119334 Moscow, Russia
[4] R. E. Alekseev Nizhny Novgorod State Technical University, Minin St. 24, 603155 Nizhny Novgorod, Russia
[5] M. V. Lomonosov Moscow State University, 1 Leninskie Gory, 119991 Moscow, Russia
[6] Departament de Química, Universitat de les Illes Balears, 07122 Palma de Mallorca, Spain
[7] Branch of Petersburg Nuclear Physics Institute Named by B.P. Konstantinov of National Research Centre «Kurchatov Institute»—Institute of Macromolecular Compounds, Bolshoi pr. VO 31, 199004 St. Petersburg, Russia
* Correspondence: nenajdenko@org.chem.msu.ru (V.G.N.); tskhovrebov-ag@rudn.ru (A.G.T.)

X-ray diffraction

Single crystals of **2a, 2c–2e** were obtained by recrystallization from dichloromethane. The intensities of reflections were collected at Bruker D8 Venture (**2c**), Bruker Apex II DUO (**2a**) diffractometers equipped with CuKα-source (λ = 1.54178 Å) or at Bruker D8 Venture (**2d**) and Bruker Apex II (**2d**) diffractometers equipped with MoKα-source (λ = 0.71073 Å). The structures were solved with a dual-space method with SHELXT [Sheldrick, G.M. (2015). Acta Cryst. A71, 3-8.] program and refined by the full-matrix least-squares technique against F^2(hkl) in anisotropic approximation for non-hydrogen atoms with SHELXL [Sheldrick, G.M. (2015). Acta Cryst. C71, 3-8.] and Olex2 [Dolomanov, O.V., Bourhis, L.J., Gildea, R.J, Howard, J.A.K. & Puschmann, H. (2009), J. Appl. Cryst. 42, 339-341.] software package. All non-hydrogen atoms were located on difference Fourier maps and refined in anisotropic approximation. The positions of hydrogen atoms were calculated, and they were refined in riding model with Uiso(H)= 1.2Ueq(C). Detailed crystallographic information for the compounds is given below.

Crystallographic information files are available from the Cambridge crystallographic Data Center upon request (https://ccdc.cam.ac.uk/structure, deposition numbers are 2408243-2408246.

Table S1. Crystallographic data and refinement parameters for **2a, 2c–2e**.

Parameter	**2a**	**2c**	**2d**	**2e**·CH_2Cl_2
Formula	$C_{12}H_8AuBrCl_2N_2Se$	$C_{12}H_7AuCl_4N_2Se$	$C_{12}H_8AuCl_2N_3O_2Se$	$C_{14}H_{10}AuCl_4N_3Se$
Fw	606.94	596.92	573.04	637.98
Crystal system, space group	Monoclinic, C2/c	Monoclinic, $P2_1/n$	Triclinic, $P\bar{1}$	Triclinic, $P\bar{1}$
a (Å)	19.8825(6)	7.0819(6)	8.1567(5)	9.194(5)
b (Å)	8.1342(2)	30.685(2)	8.4866(5)	9.970(9)
c (Å)	19.9588(6)	14.1901(11)	11.7124(8)	10.424(6)
α (°)	90	90	106.050(2)	83.27(4)
β (°)	111.929(1)	101.011(3)	93.208(2)	78.768(19)
γ (°)	90	90	103.869(2)	83.05(5)
V ($Å^3$)	2994.35(15)	3026.8(4)	749.92(8)	926.0(11)
Z	8	8	2	2
μ (cm^{-1})	27.548	27.432	12.597	10.485
D_{calc} (g cm^{-3})	2.693	2.620	2.538	2.288
No. of meas., indep. and obsvd. [$I > 2\sigma(I)$] reflns	19756, 2551, 2331	20838, 5865, 5156	10766, 4601, 3681	11120, 6321, 5509
R_{int}	0.069	0.078	0.043	0.023
$R[F^2 > 2\sigma(F^2)]$, $wR(F^2)$, S	0.044, 0.135, 1.112	0.055, 0.151, 1.077	0.038, 0.088, 1.071	0.046, 0.121, 1.036
$\Delta\rho_{max}$, $\Delta\rho_{min}$ (e $Å^{-3}$)	1.686, -1.481	5.399, -2.565	2.944, -1.919	9.119, - 1.328

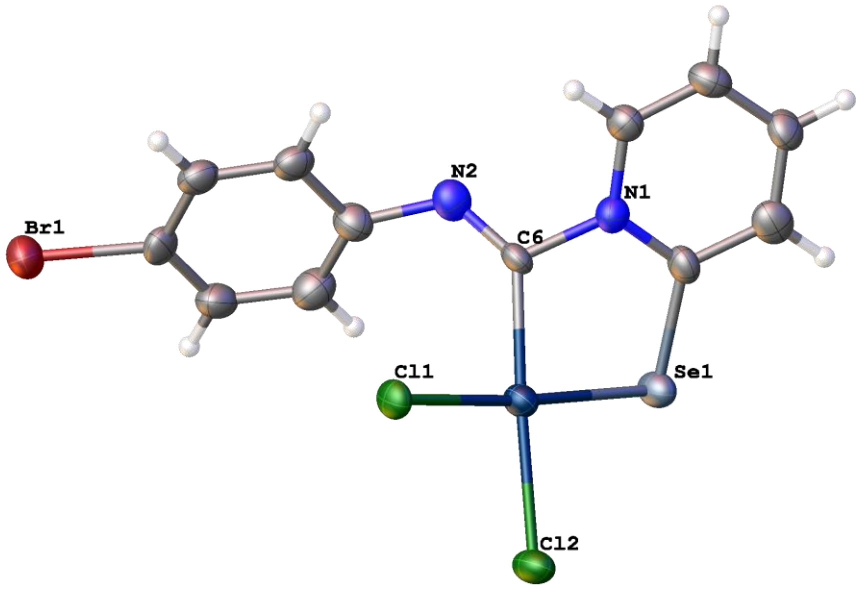

Figure S1. Molecular view of **2a** in representation of atoms with thermal ellipsoids.

Table S2. Bond Lengths for **2a**.

**Atom**	**Atom**	**Length/Å**	**Atom**	**Atom**	**Length/Å**
Au1	Se1	2.4060(9)	C1	C2	1.394(12)
Au1	Cl1	2.3439(18)	C2	C3	1.366(13)
Au1	Cl2	2.357(2)	C3	C4	1.398(14)
Au1	C6	2.021(9)	C4	C5	1.354(13)
Br1	C10	1.911(8)	C7	C8	1.393(13)
Se1	C1	1.884(9)	C7	C12	1.385(15)
N1	C1	1.348(11)	C8	C9	1.358(13)
N1	C5	1.368(11)	C9	C10	1.391(15)
N1	C6	1.481(10)	C10	C11	1.370(13)
N2	C6	1.239(12)	C11	C12	1.378(14)
N2	C7	1.427(11)			

Table S3. Bond Angles for **2a**.

**Atom**	**Atom**	**Atom**	**Angle/°**	**Atom**	**Atom**	**Atom**	**Angle/°**
Cl1	Au1	Se1	174.61(5)	C5	C4	C3	119.5(8)
Cl1	Au1	Cl2	89.93(7)	C4	C5	N1	119.9(8)
Cl2	Au1	Se1	93.75(6)	N1	C6	Au1	113.2(6)
C6	Au1	Se1	83.2(2)	N2	C6	Au1	133.8(6)
C6	Au1	Cl1	93.1(2)	N2	C6	N1	113.0(7)
C6	Au1	Cl2	176.9(2)	C8	C7	N2	115.6(8)
C1	Se1	Au1	92.3(2)	C12	C7	N2	124.3(8)
C1	N1	C5	121.9(7)	C12	C7	C8	119.7(9)
C1	N1	C6	118.5(7)	C9	C8	C7	120.3(9)
C5	N1	C6	119.4(7)	C8	C9	C10	118.8(8)
C6	N2	C7	125.7(8)	C9	C10	Br1	117.6(7)
N1	C1	Se1	117.6(6)	C11	C10	Br1	120.4(8)
N1	C1	C2	118.9(9)	C11	C10	C9	121.9(8)
C2	C1	Se1	123.5(7)	C10	C11	C12	118.7(9)
C3	C2	C1	119.8(9)	C11	C12	C7	120.2(9)
C2	C3	C4	119.9(8)				

Table S4. Torsion Angles for **2a**.

A	B	C	D	Angle/°	A	B	C	D	Angle/°
Au1	Se1	C1	N1	-20.9(7)	C5	N1	C6	N2	32.8(11)
Au1	Se1	C1	C2	159.2(8)	C6	N1	C1	Se1	-3.4(10)
Br1	C10	C11	C12	178.5(8)	C6	N1	C1	C2	176.6(8)
Se1	C1	C2	C3	177.8(7)	C6	N1	C5	C4	-174.6(8)
N1	C1	C2	C3	-2.2(13)	C6	N2	C7	C8	134.1(10)
N2	C7	C8	C9	179.3(8)	C6	N2	C7	C12	-53.6(15)
N2	C7	C12	C11	-175.5(9)	C7	N2	C6	Au1	0.0(14)
C1	N1	C5	C4	1.7(13)	C7	N2	C6	N1	176.2(8)
C1	N1	C6	Au1	33.3(9)	C7	C8	C9	C10	-5.8(14)
C1	N1	C6	N2	-143.7(8)	C8	C7	C12	C11	-3.5(16)
C1	C2	C3	C4	2.3(14)	C8	C9	C10	Br1	-175.5(7)
C2	C3	C4	C5	-0.4(14)	C8	C9	C10	C11	2.0(15)
C3	C4	C5	N1	-1.6(14)	C9	C10	C11	C12	1.0(15)
C5	N1	C1	Se1	-179.8(6)	C10	C11	C12	C7	-0.2(16)
C5	N1	C1	C2	0.2(13)	C12	C7	C8	C9	6.6(15)
C5	N1	C6	Au1	-150.2(6)					

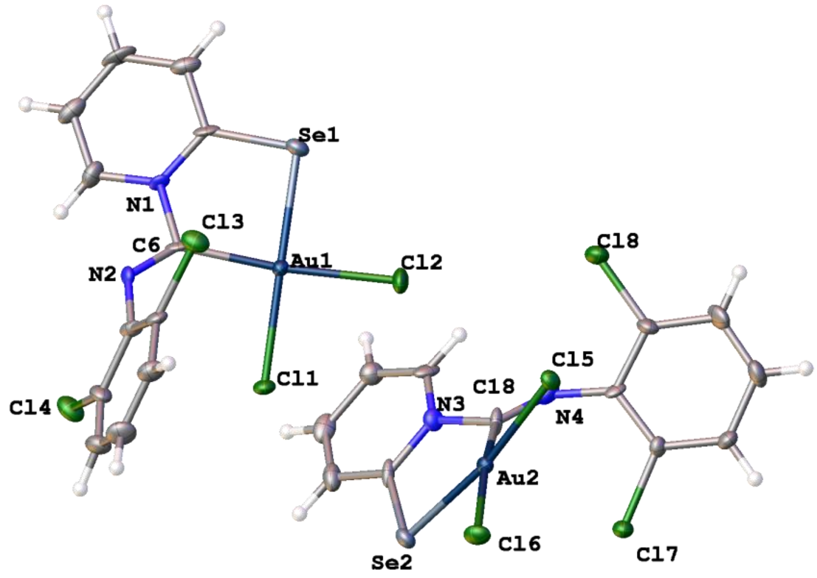

Figure S2. Molecular view of **2c** in representation of atoms with thermal ellipsoids.

Table S5. Bond Lengths for **2c**.

Atom	Atom	Length/Å	Atom	Atom	Length/Å
Au1	Se1	2.3699(15)	Au2	Se2	2.3866(15)
Au1	Cl1	2.330(3)	Au2	Cl5	2.337(3)
Au1	Cl2	2.344(3)	Au2	Cl6	2.354(3)
Au1	C6	2.024(13)	Au2	C18	1.983(13)
Se1	C1	1.869(13)	Se2	C13	1.873(14)
N1	C1	1.356(18)	N3	C13	1.361(19)
N1	C5	1.367(17)	N3	C17	1.350(18)
N1	C6	1.473(14)	N3	C18	1.493(15)
C1	C2	1.401(17)	N4	C18	1.285(18)
N2	C6	1.267(18)	N4	C19	1.368(16)
N2	C7	1.372(16)	Cl7	C20	1.736(14)
C2	C3	1.37(2)	Cl8	C24	1.727(13)
Cl3	C8	1.721(15)	C13	C14	1.401(18)
C3	C4	1.39(2)	C14	C15	1.38(2)
Cl4	C12	1.719(15)	C15	C16	1.40(2)
C4	C5	1.395(19)	C16	C17	1.377(19)
C7	C8	1.397(19)	C19	C20	1.403(16)
C7	C12	1.43(2)	C19	C24	1.399(19)
C8	C9	1.412(19)	C20	C21	1.375(18)
C9	C10	1.36(2)	C21	C22	1.41(2)
C10	C11	1.37(2)	C22	C23	1.36(2)
C11	C12	1.38(2)	C23	C24	1.40(2)

Table S6. Bond Angles for **2c**.

Atom	Atom	Atom	Angle/°	Atom	Atom	Atom	Angle/°
Cl1	Au1	Se1	173.80(9)	Cl5	Au2	Se2	171.29(8)
Cl1	Au1	Cl2	91.50(12)	Cl5	Au2	Cl6	93.60(11)
Cl2	Au1	Se1	88.62(10)	Cl6	Au2	Se2	90.51(10)
C6	Au1	Se1	86.1(4)	C18	Au2	Se2	83.6(4)
C6	Au1	Cl1	94.2(4)	C18	Au2	Cl5	93.4(4)
C6	Au1	Cl2	173.2(4)	C18	Au2	Cl6	169.8(4)
C1	Se1	Au1	94.2(4)	C13	Se2	Au2	93.4(4)
C1	N1	C5	122.5(11)	C13	N3	C18	115.8(11)
C1	N1	C6	118.6(11)	C17	N3	C13	123.2(12)
C5	N1	C6	118.5(12)	C17	N3	C18	120.7(12)
N1	C1	Se1	119.4(9)	C18	N4	C19	128.3(11)
N1	C1	C2	119.3(13)	N3	C13	Se2	118.7(9)
C2	C1	Se1	121.3(11)	N3	C13	C14	117.8(13)
C6	N2	C7	126.7(11)	C14	C13	Se2	123.4(12)
C3	C2	C1	119.3(15)	C15	C14	C13	119.8(15)
C2	C3	C4	120.8(12)	C14	C15	C16	120.6(13)
C3	C4	C5	119.2(14)	C17	C16	C15	118.3(13)
N1	C5	C4	118.8(15)	N3	C17	C16	120.3(14)
N1	C6	Au1	115.0(9)	N3	C18	Au2	116.3(9)
N2	C6	Au1	130.8(9)	N4	C18	Au2	131.5(9)
N2	C6	N1	114.0(11)	N4	C18	N3	112.2(11)
N2	C7	C8	121.5(13)	N4	C19	C20	125.8(12)
N2	C7	C12	120.6(13)	N4	C19	C24	117.1(11)
C8	C7	C12	117.3(12)	C24	C19	C20	116.0(12)
C7	C8	Cl3	119.8(10)	C19	C20	Cl7	120.0(10)
C7	C8	C9	120.4(13)	C21	C20	Cl7	117.3(10)
C9	C8	Cl3	119.8(11)	C21	C20	C19	122.7(13)
C10	C9	C8	121.0(14)	C20	C21	C22	118.3(12)
C9	C10	C11	119.3(14)	C23	C22	C21	121.8(13)
C10	C11	C12	121.8(15)	C22	C23	C24	118.1(14)
C7	C12	Cl4	118.4(11)	C19	C24	Cl8	117.6(10)
C11	C12	Cl4	121.4(12)	C19	C24	C23	123.0(13)
C11	C12	C7	120.1(14)	C23	C24	Cl8	119.2(11)

Table S7. Torsion Angles for **2c**.

A	B	C	D	Angle/°	A	B	C	D	Angle/°
Au1	Se1	C1	N1	7.4(10)	Au2	Se2	C13	N3	17.1(10)
Au1	Se1	C1	C2	-172.9(10)	Au2	Se2	C13	C14	-164.9(11)
Se1	C1	C2	C3	177.7(10)	Se2	C13	C14	C15	-177.6(10)
N1	C1	C2	C3	-2.7(19)	N3	C13	C14	C15	0.5(19)
C1	N1	C5	C4	-1.0(19)	N4	C19	C20	Cl7	-12.4(18)
C1	N1	C6	Au1	-27.3(13)	N4	C19	C20	C21	168.3(12)
C1	N1	C6	N2	147.8(12)	N4	C19	C24	Cl8	14.6(16)
C1	C2	C3	C4	0(2)	N4	C19	C24	C23	-169.3(13)
N2	C7	C8	Cl3	-9.5(17)	Cl7	C20	C21	C22	179.3(10)
N2	C7	C8	C9	171.2(12)	C13	N3	C17	C16	0(2)
N2	C7	C12	Cl4	9.6(17)	C13	N3	C18	Au2	-31.6(14)
N2	C7	C12	C11	-173.2(12)	C13	N3	C18	N4	147.9(12)
C2	C3	C4	C5	2(2)	C13	C14	C15	C16	-1(2)
Cl3	C8	C9	C10	-179.2(11)	C14	C15	C16	C17	2(2)
C3	C4	C5	N1	-2(2)	C15	C16	C17	N3	-1(2)
C5	N1	C1	Se1	-177.1(10)	C17	N3	C13	Se2	178.3(10)
C5	N1	C1	C2	3.2(18)	C17	N3	C13	C14	0.1(18)
C5	N1	C6	Au1	160.1(9)	C17	N3	C18	Au2	154.6(10)
C5	N1	C6	N2	-24.7(16)	C17	N3	C18	N4	-25.8(17)
C6	N1	C1	Se1	10.7(15)	C18	N3	C13	Se2	4.7(14)
C6	N1	C1	C2	-169.0(11)	C18	N3	C13	C14	-173.5(11)
C6	N1	C5	C4	171.2(11)	C18	N3	C17	C16	173.4(12)
C6	N2	C7	C8	71.0(18)	C18	N4	C19	C20	60.7(19)
C6	N2	C7	C12	-118.0(15)	C18	N4	C19	C24	-132.2(15)
C7	N2	C6	Au1	6(2)	C19	N4	C18	Au2	3(2)
C7	N2	C6	N1	-168.6(12)	C19	N4	C18	N3	-176.6(11)
C7	C8	C9	C10	0(2)	C19	C20	C21	C22	-1(2)
C8	C7	C12	Cl4	-179.0(10)	C20	C19	C24	Cl8	-177.0(9)
C8	C7	C12	C11	-1.8(19)	C20	C19	C24	C23	-1(2)
C8	C9	C10	C11	2(2)	C20	C21	C22	C23	2(2)
C9	C10	C11	C12	-4(2)	C21	C22	C23	C24	-1(2)
C10	C11	C12	Cl4	-179.0(12)	C22	C23	C24	Cl8	177.2(11)
C10	C11	C12	C7	4(2)	C22	C23	C24	C19	1(2)
C12	C7	C8	Cl3	179.2(9)	C24	C19	C20	Cl7	-179.6(10)
C12	C7	C8	C9	-0.1(18)	C24	C19	C20	C21	1.1(19)

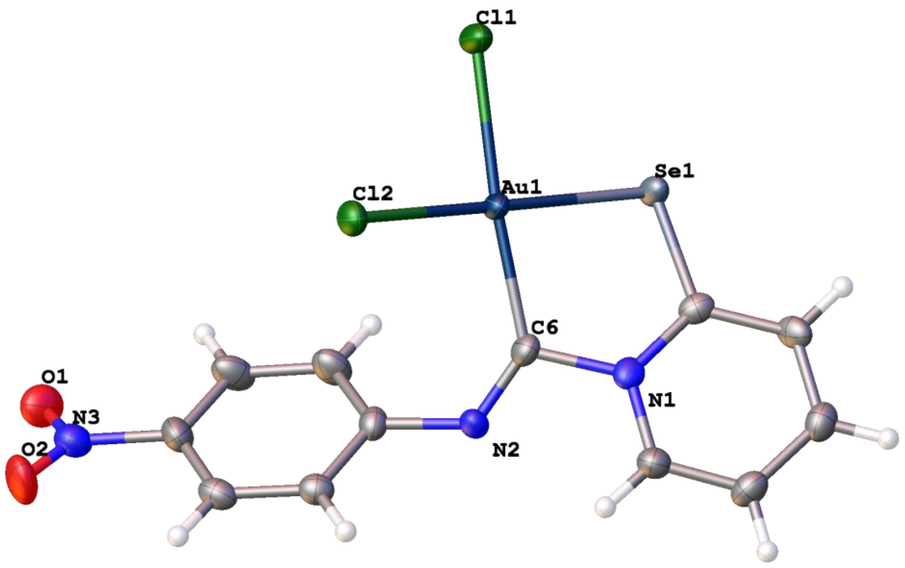

Figure S3. Molecular view of **2d** in representation of atoms with thermal ellipsoids.

Table S8. Bond Lengths for **2d**.

Atom	Atom	Length/Å	Atom	Atom	Length/Å
Au1	Se1	2.3910(6)	N2	C7	1.421(7)
Au1	Cl1	2.3371(15)	C2	C3	1.372(8)
Au1	Cl2	2.3427(13)	N3	C10	1.459(8)
Au1	C6	2.020(5)	C3	C4	1.410(8)
Se1	C1	1.881(5)	C4	C5	1.365(7)
O1	N3	1.222(7)	C7	C8	1.393(7)
N1	C1	1.353(7)	C7	C12	1.393(8)
N1	C5	1.371(7)	C8	C9	1.377(8)
N1	C6	1.472(7)	C9	C10	1.400(8)
C1	C2	1.390(8)	C10	C11	1.364(8)
O2	N3	1.234(7)	C11	C12	1.382(8)
N2	C6	1.247(7)			

Table S9. Bond Angles for **2d**.

Atom	Atom	Atom	Angle/°	Atom	Atom	Atom	Angle/°
Cl1	Au1	Se1	88.57(4)	O2	N3	C10	118.7(6)
Cl1	Au1	Cl2	91.84(5)	C2	C3	C4	119.5(5)
Cl2	Au1	Se1	172.44(4)	C5	C4	C3	119.3(5)
C6	Au1	Se1	84.47(15)	C4	C5	N1	120.1(5)
C6	Au1	Cl1	171.80(15)	N1	C6	Au1	114.0(4)
C6	Au1	Cl2	95.63(15)	N2	C6	Au1	133.0(4)
C1	Se1	Au1	92.46(17)	N2	C6	N1	113.0(5)
C1	N1	C5	121.6(5)	C8	C7	N2	116.4(5)
C1	N1	C6	119.0(4)	C8	C7	C12	120.0(5)
C5	N1	C6	119.2(5)	C12	C7	N2	123.5(5)
N1	C1	Se1	118.1(4)	C9	C8	C7	120.3(5)
N1	C1	C2	119.4(5)	C8	C9	C10	118.4(5)
C2	C1	Se1	122.4(4)	C9	C10	N3	118.2(5)
C6	N2	C7	125.6(5)	C11	C10	N3	120.1(6)
C3	C2	C1	120.1(5)	C11	C10	C9	121.7(6)
O1	N3	O2	123.6(6)	C10	C11	C12	119.8(6)
O1	N3	C10	117.6(5)	C11	C12	C7	119.6(5)

Table S10. Torsion Angles for **2d**.

A	B	C	D	Angle/°	A	B	C	D	Angle/°
Au1	Se1	C1	N1	20.4(4)	C5	N1	C1	C2	-1.1(8)
Au1	Se1	C1	C2	-163.2(5)	C5	N1	C6	Au1	157.1(4)
Se1	C1	C2	C3	-175.0(4)	C5	N1	C6	N2	-25.0(7)
O1	N3	C10	C9	-164.3(6)	C6	N1	C1	Se1	0.6(6)
O1	N3	C10	C11	15.4(9)	C6	N1	C1	C2	-175.9(5)
N1	C1	C2	C3	1.4(8)	C6	N1	C5	C4	174.6(5)
C1	N1	C5	C4	-0.2(8)	C6	N2	C7	C8	-135.4(6)
C1	N1	C6	Au1	-27.9(6)	C6	N2	C7	C12	49.2(9)
C1	N1	C6	N2	149.9(5)	C7	N2	C6	Au1	1.6(9)
C1	C2	C3	C4	-0.5(9)	C7	N2	C6	N1	-175.7(5)
O2	N3	C10	C9	15.8(9)	C7	C8	C9	C10	3.3(8)
O2	N3	C10	C11	-164.5(6)	C8	C7	C12	C11	3.6(9)
N2	C7	C8	C9	179.6(5)	C8	C9	C10	N3	179.2(5)
N2	C7	C12	C11	178.8(6)	C8	C9	C10	C11	-0.4(9)
C2	C3	C4	C5	-0.8(9)	C9	C10	C11	C12	-0.9(10)
N3	C10	C11	C12	179.5(6)	C10	C11	C12	C7	-0.7(10)
C3	C4	C5	N1	1.2(9)	C12	C7	C8	C9	-4.9(8)
C5	N1	C1	Se1	175.5(4)					

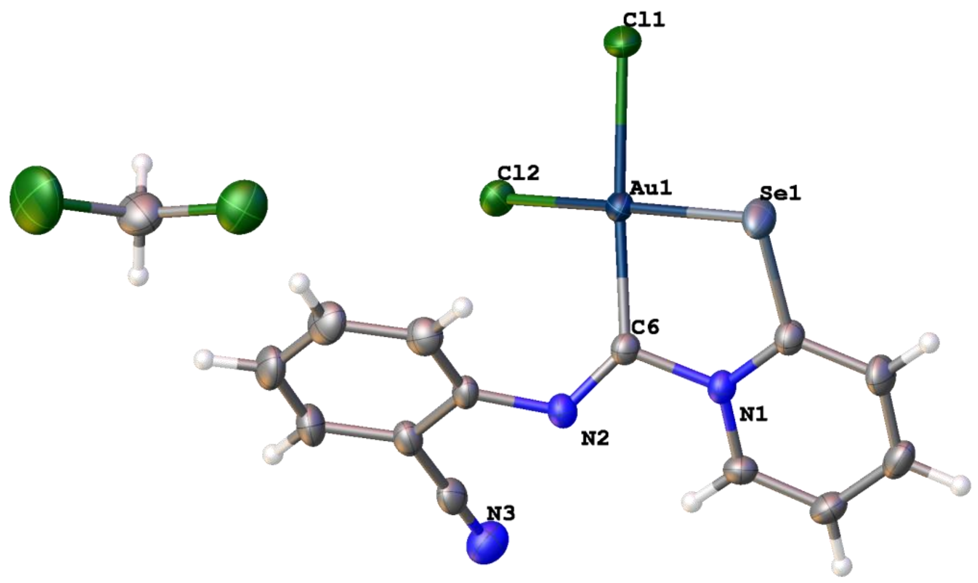

Figure S4. Molecular view of **2e** in representation of atoms with thermal ellipsoids.

Table S11. Bond Lengths for **2e**.

**Atom**	**Atom**	**Length/Å**	**Atom**	**Atom**	**Length/Å**
Au1	Se1	2.3878(16)	C2	C3	1.371(10)
Au1	Cl1	2.349(2)	C3	C4	1.392(10)
Au1	Cl2	2.333(2)	C4	C5	1.362(9)
Au1	C6	2.022(6)	C7	C8	1.386(9)
Se1	C1	1.877(6)	C7	C12	1.404(8)
N1	C1	1.356(7)	C8	C9	1.398(10)
N1	C5	1.357(8)	C9	C10	1.390(11)
N1	C6	1.473(7)	C10	C11	1.400(11)
N2	C6	1.257(7)	C11	C12	1.390(9)
N2	C7	1.403(7)	C12	C13	1.444(9)
N3	C13	1.157(9)	Cl3	C14	1.751(10)
C1	C2	1.403(8)	Cl4	C14	1.770(9)

Table S12. Bond Angles for **2e**.

Atom	Atom	Atom	Angle/°	Atom	Atom	Atom	Angle/°
Cl1	Au1	Se1	90.04(8)	N1	C5	C4	120.8(6)
Cl2	Au1	Se1	171.51(5)	N1	C6	Au1	114.0(4)
Cl2	Au1	Cl1	91.78(9)	N2	C6	Au1	132.0(4)
C6	Au1	Se1	84.31(17)	N2	C6	N1	114.0(5)
C6	Au1	Cl1	173.94(16)	N2	C7	C12	117.0(5)
C6	Au1	Cl2	94.13(17)	C8	C7	N2	123.7(6)
C1	Se1	Au1	92.99(19)	C8	C7	C12	119.1(5)
C1	N1	C5	121.8(5)	C7	C8	C9	120.2(6)
C1	N1	C6	118.1(5)	C10	C9	C8	120.4(7)
C5	N1	C6	120.1(5)	C9	C10	C11	120.0(6)
C6	N2	C7	124.7(5)	C12	C11	C10	119.1(7)
N1	C1	Se1	118.6(4)	C7	C12	C13	118.3(5)
N1	C1	C2	118.7(6)	C11	C12	C7	121.1(6)
C2	C1	Se1	122.6(5)	C11	C12	C13	120.5(6)
C3	C2	C1	119.1(6)	N3	C13	C12	179.9(7)
C2	C3	C4	121.0(6)	Cl3	C14	Cl4	112.0(5)
C5	C4	C3	118.5(6)				

Table S13. Torsion Angles for **2e**.

A	B	C	D	Angle/°	A	B	C	D	Angle/°
Au1	Se1	C1	N1	-16.5(5)	C5	N1	C6	N2	32.1(8)
Au1	Se1	C1	C2	160.6(6)	C6	N1	C1	Se1	-6.0(7)
Se1	C1	C2	C3	-175.5(5)	C6	N1	C1	C2	176.9(6)
N1	C1	C2	C3	1.6(10)	C6	N1	C5	C4	-177.6(6)
N2	C7	C8	C9	-178.6(6)	C6	N2	C7	C8	-53.0(9)
N2	C7	C12	C11	179.3(6)	C6	N2	C7	C12	131.3(6)
N2	C7	C12	C13	-4.0(8)	C7	N2	C6	Au1	-2.0(9)
C1	N1	C5	C4	1.7(10)	C7	N2	C6	N1	176.7(5)
C1	N1	C6	Au1	31.7(6)	C7	C8	C9	C10	2.1(10)
C1	N1	C6	N2	-147.2(6)	C8	C7	C12	C11	3.4(9)
C1	C2	C3	C4	0.0(11)	C8	C7	C12	C13	-179.9(6)
C2	C3	C4	C5	-0.9(11)	C8	C9	C10	C11	-1.4(11)
C3	C4	C5	N1	0.0(10)	C9	C10	C11	C12	1.8(11)
C5	N1	C1	Se1	174.7(5)	C10	C11	C12	C7	-2.8(10)
C5	N1	C1	C2	-2.4(9)	C10	C11	C12	C13	-179.4(6)
C5	N1	C6	Au1	-149.0(5)	C12	C7	C8	C9	-3.0(9)

NMR spectra

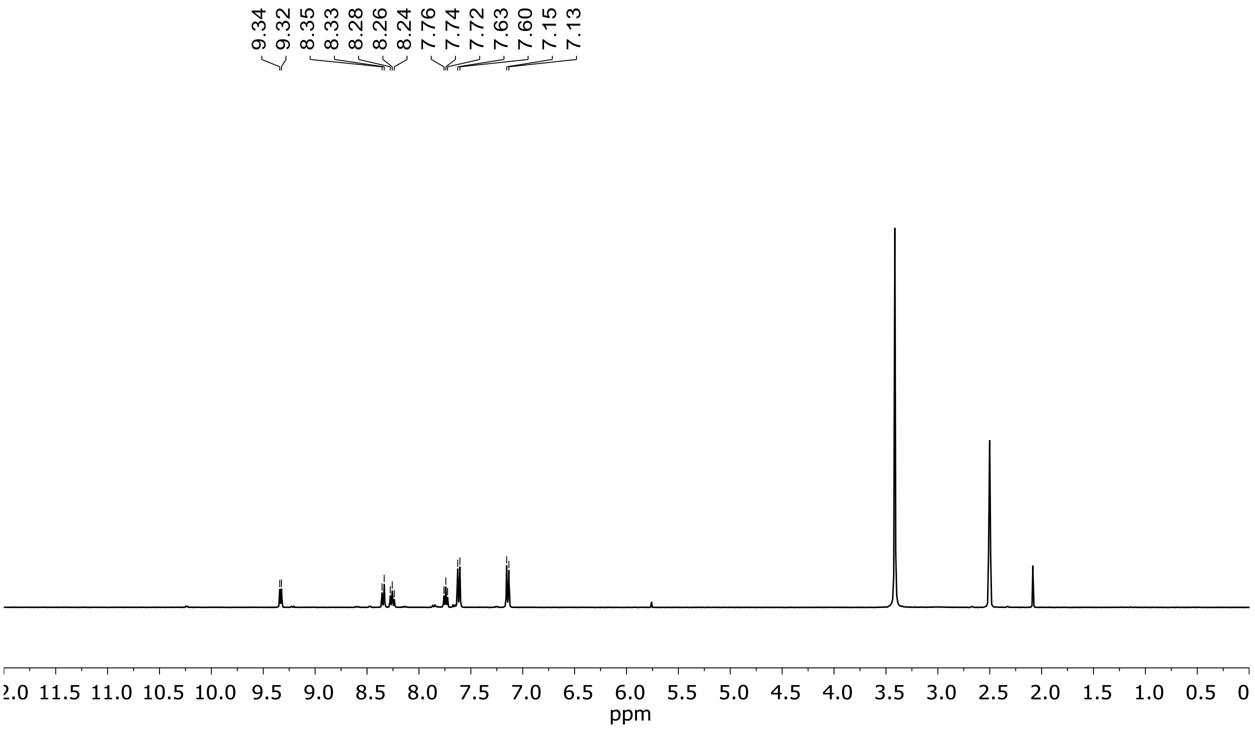

^{1}H NMR spectrum (400 MHz, DMSO-*d6*) of **2a**

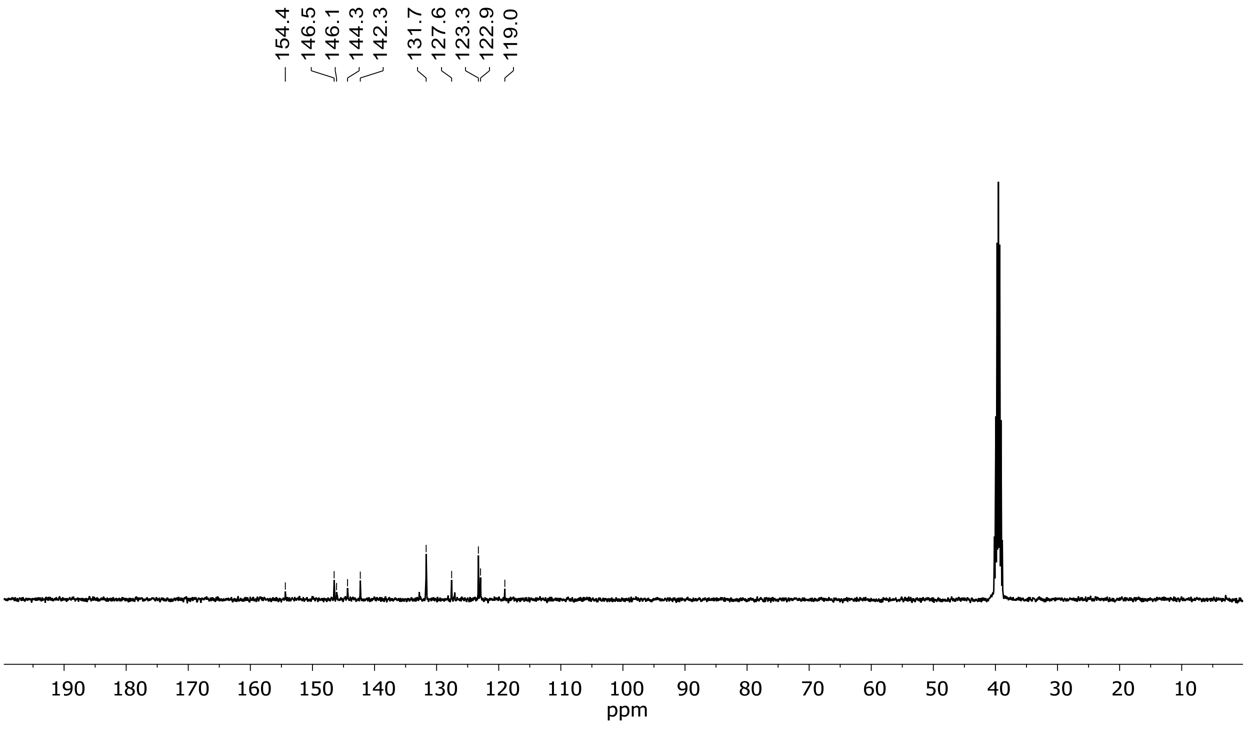

^{13}C{H} NMR spectrum (101 MHz, DMSO-*d6*) of **2a**

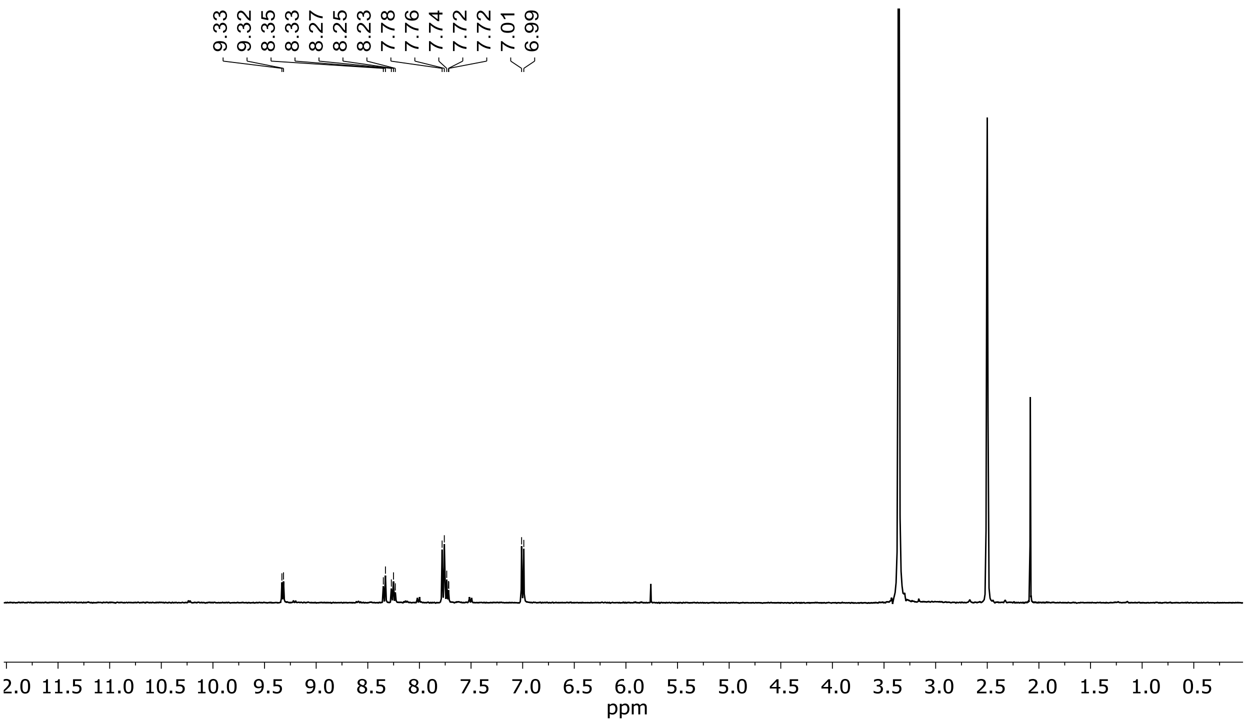

^{1}H NMR spectrum (400 MHz, DMSO-*d6*) of **2b**

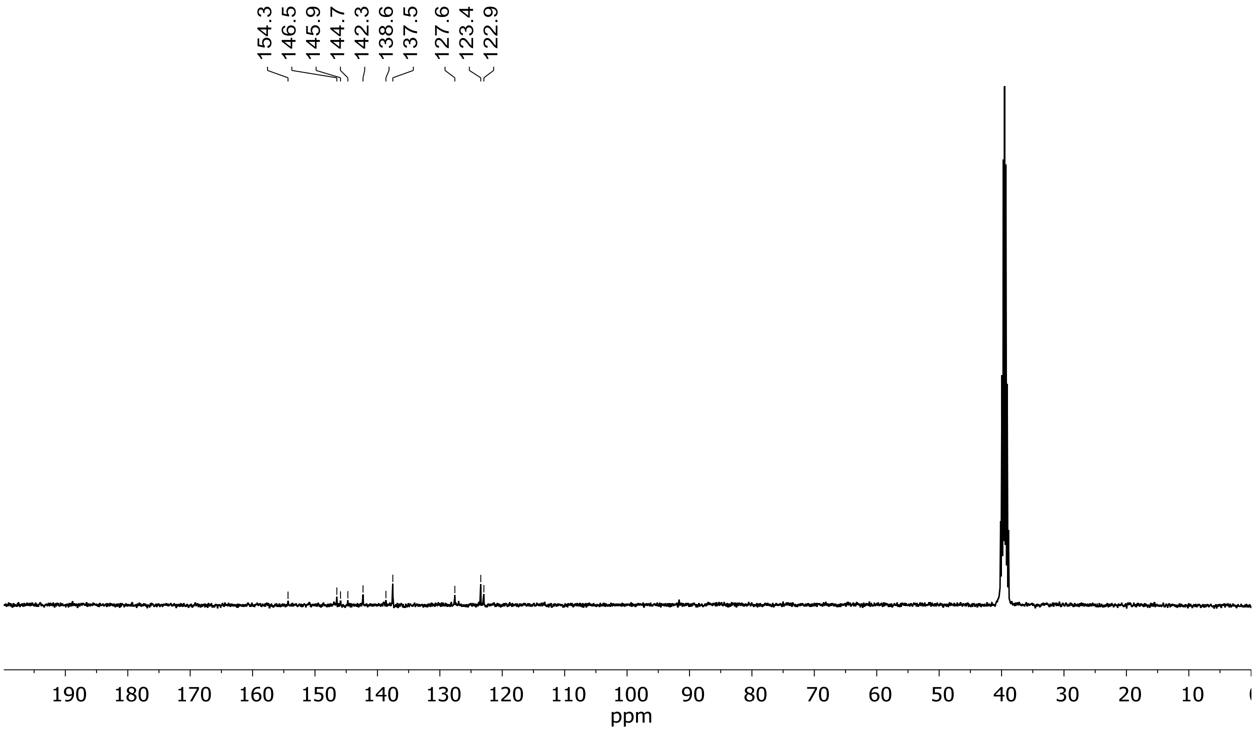

^{13}C{H} NMR spectrum (101 MHz, DMSO-*d6*) of **2b**

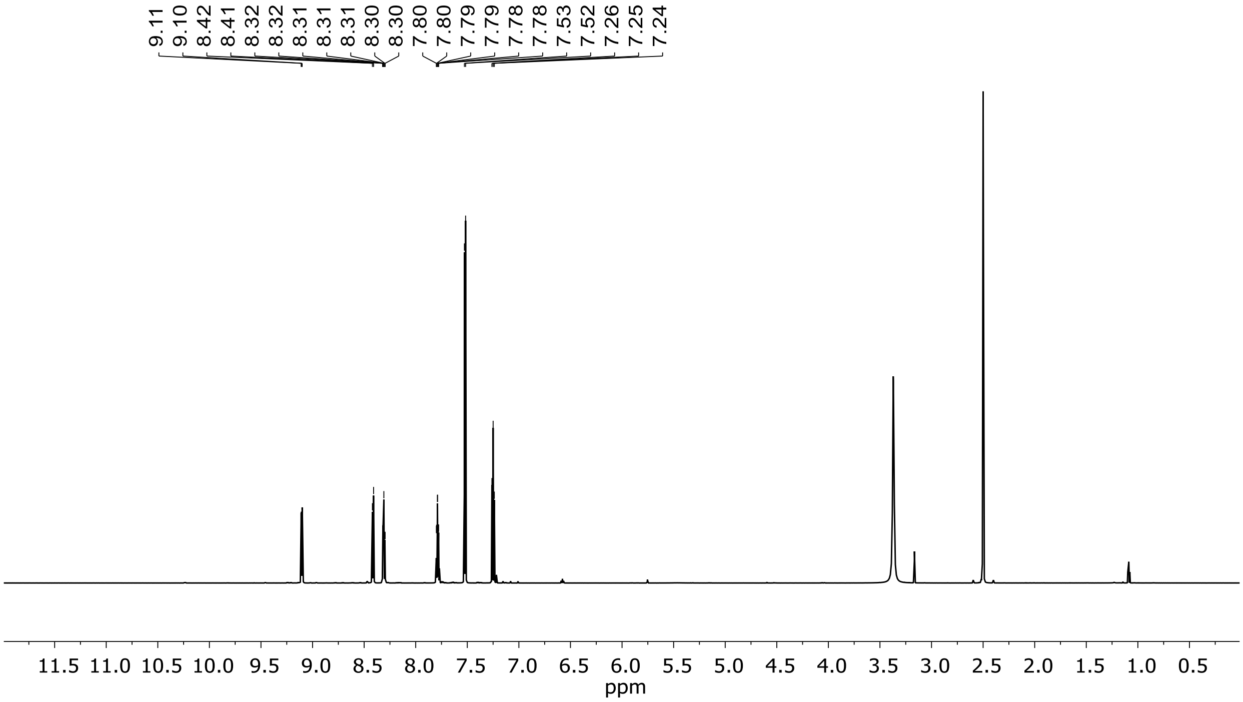

^{1}H NMR spectrum (700 MHz, DMSO-*d6*) of **2c**

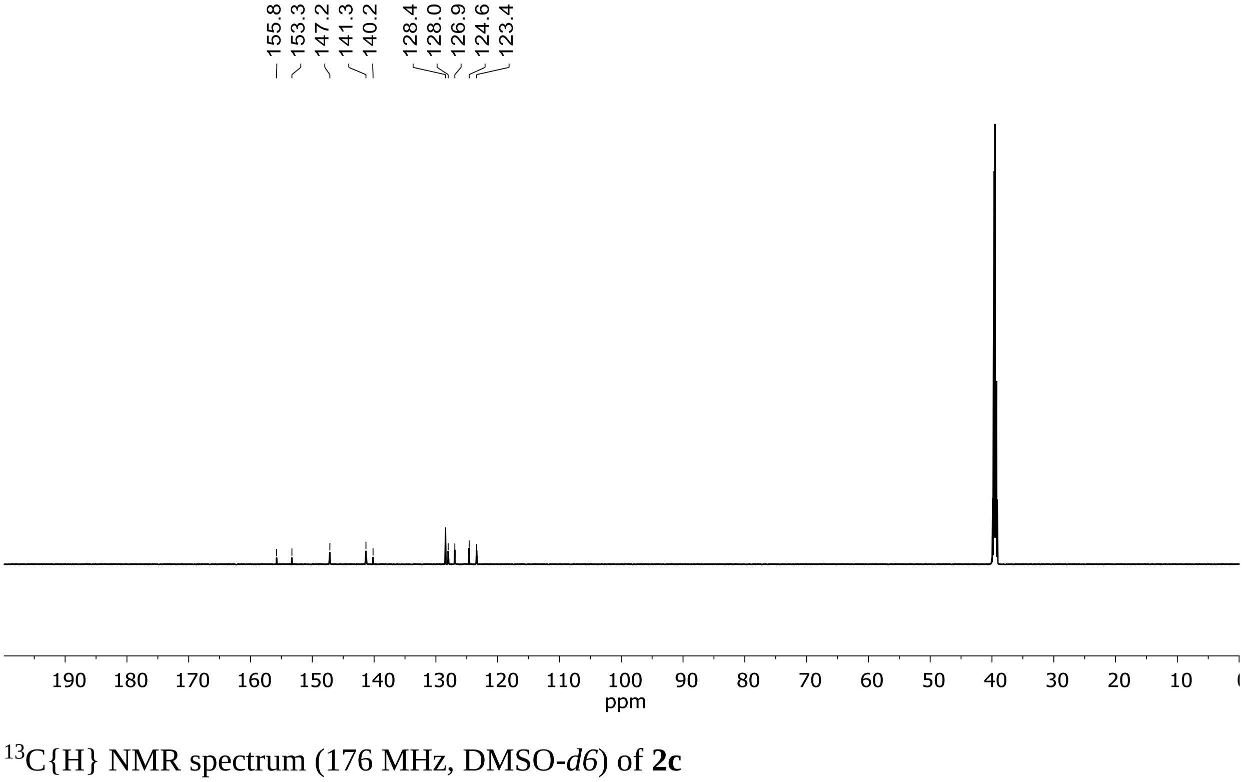

^{13}C{H} NMR spectrum (176 MHz, DMSO-*d6*) of **2c**

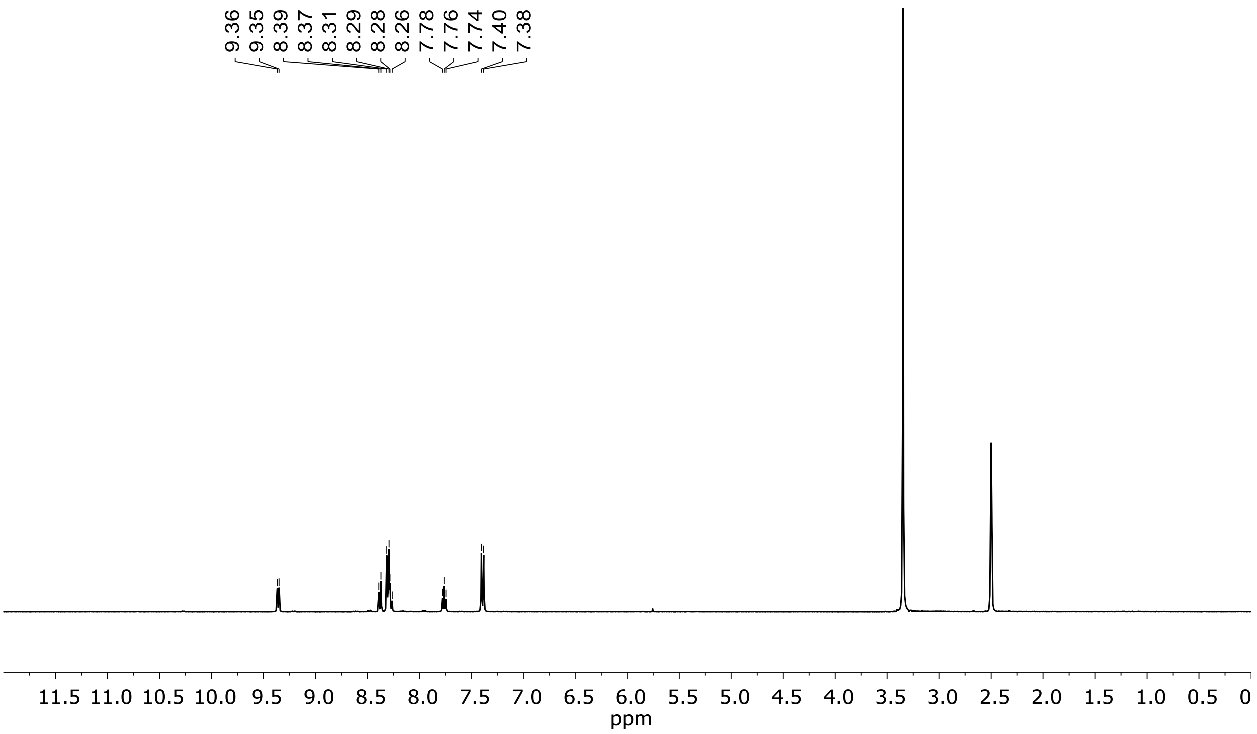

^{1}H NMR spectrum (400 MHz, DMSO-*d6*) of **2d**

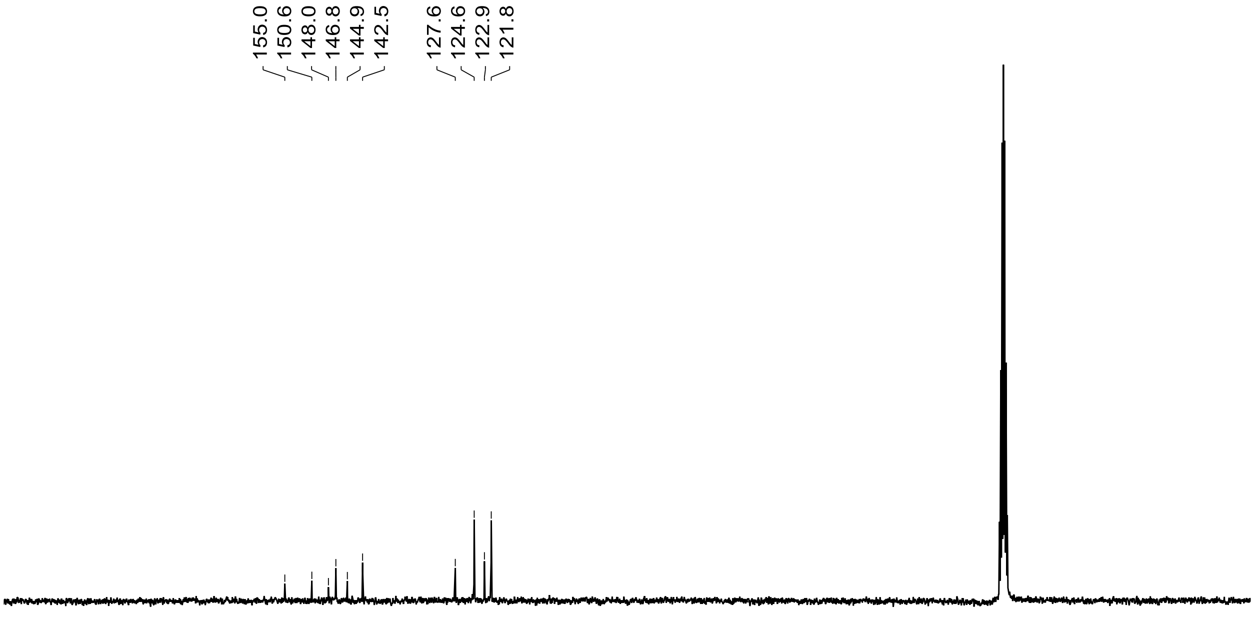

^{13}C{H} NMR spectrum (101 MHz, DMSO-*d6*) of **2d**

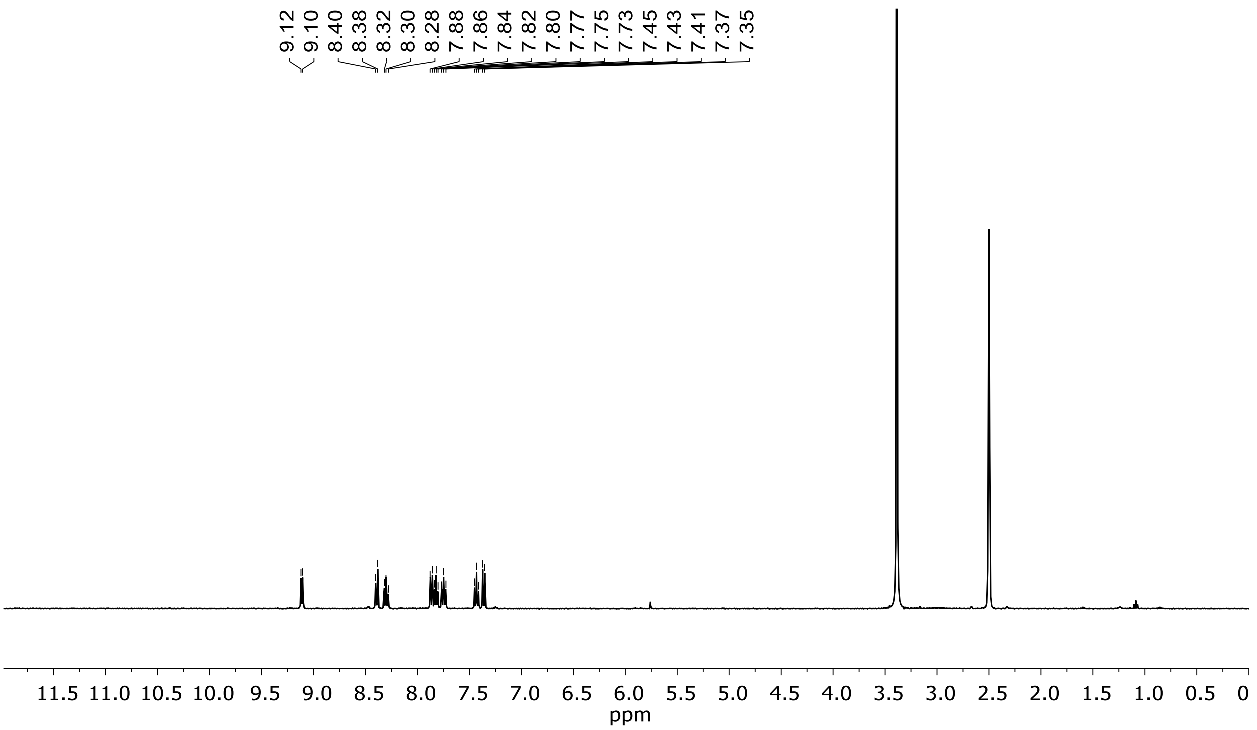

^{1}H NMR spectrum (400 MHz, DMSO-*d6*) of **2e**

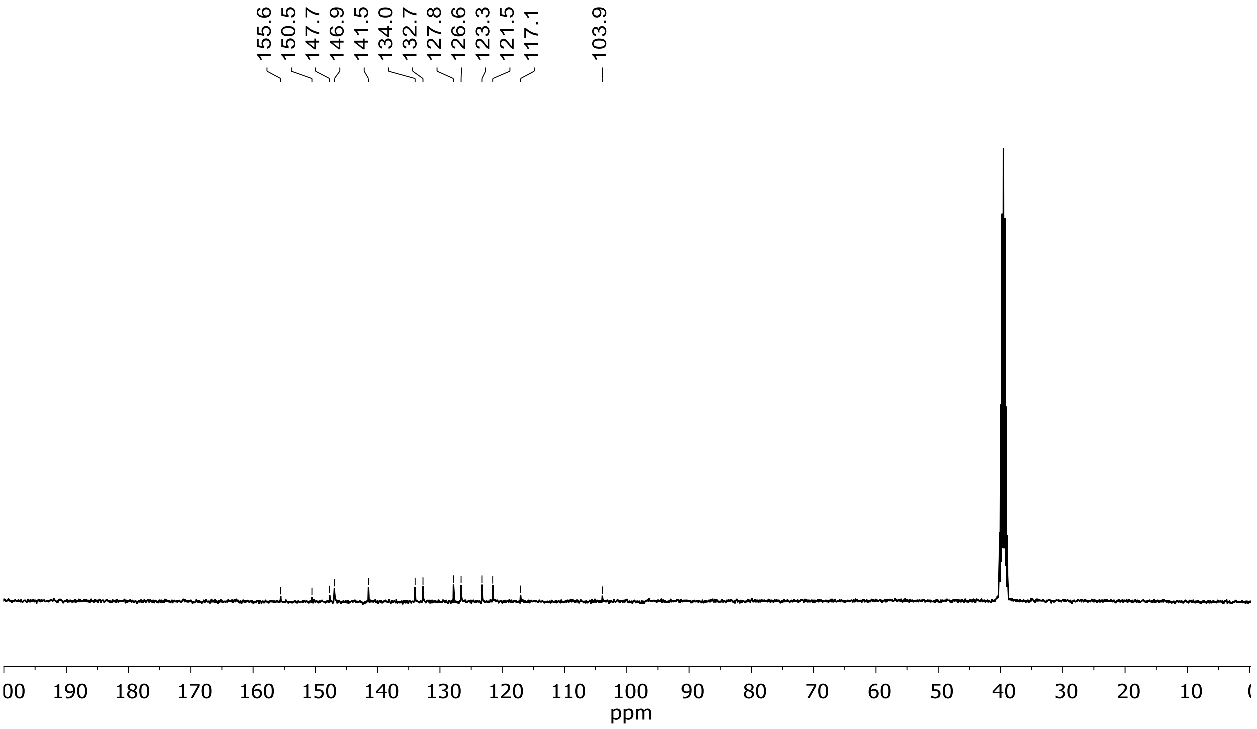

$^{13}C\{H\}$ NMR spectrum (101 MHz, DMSO-*d6*) of **2e**
